# Self-care of chronic musculoskeletal pain – experiences and attitudes of patients and health care providers

**DOI:** 10.1186/s12891-018-1997-7

**Published:** 2018-03-07

**Authors:** Irena Kovačević, Višnja Majerić Kogler, Tihana Magdić Turković, Lidija Fumić Dunkić, Željko Ivanec, Davorina Petek

**Affiliations:** 1 University of Applied Health Sciences, Mlinarska 38, 10 000 Zagreb, Croatia; 2 School of Medicine, University of Zagreb, Šalata 3, Zagreb, Croatia; 3 Sisters of Charity University Hospital Centre, Vinogradska cesta 29, Zagreb, Croatia; 4 Department of Family Medicine, Faculty of Medicine, University of Ljubljana, Poljanski nasip 58, 1000 Ljubljana, Slovenia

**Keywords:** Self-care, Chronic musculoskeletal pain, Attitudes, Patients, Health care providers, Qualitative study

## Abstract

**Background:**

Self-care is often the first choice for people with chronic musculoskeletal pain. Self-care includes the use of non-prescription medications with no doctor’s supervision, as well as the use of other modern and traditional treatment methods with no consultation of the health care provider. Self-care may have positive effects on the successful outcome of a multidisciplinary approach to treatment. The aim of this study was to investigate the experiences and attitudes of patients and health care providers to the self-care of chronic musculoskeletal pain.

**Methods:**

Qualitative Phenomenological study, where the data were collected by the method of an audio-taped interview in 15 patients at the outpatient clinic for pain management and in 20 health care providers involved in the treatment of those patients. The interviews were transcribed verbatim and analyzed by principles of Interpretative Thematic Analysis.

**Results:**

Topics identified in patients: a) positive aspects of self-care, b) a need for pain self-care, c) social aspects of pain self-care. Topics identified in health care providers: a) aspects of self-care, b) a need for self-care c) risks of self-care.

Most of patients have positive attitude to self-care and this is the first step to pain management and to care for itself. The most frequent factors influencing decision about the self-care are heavy pain, unavailability of the doctor, long awaiting time for the therapy, or ineffectiveness of methods of conventional medicine. The health care providers believe that self-care of chronic musculoskeletal pain may be a patient’s contribution to clinical treatment. However, good awareness of methods used is important in this context, to avoid adverse effects of self-care.

**Conclusion:**

Patients understand the self-care of musculoskeletal pain as an individually adjusted treatment and believe in its effectiveness. Health care providers support self-care as an adjunction to clinical management only, and think that self-care of musculoskeletal pain acts as a placebo, with a short-lived effect on chronic musculoskeletal pain.

## Background

Due to its unique neurobiological ground and the influence on physical function, quality of life and medical outcome, chronic pain can be considered chronic disease [1]. The prevalence of pain varies depending on the age, life style and general health in the population [2]. The incidence of pain doubles around the age of sixty and has the tendency to further increase with each decade of life [3].

The chronic pain has been recognized as one that lasts longer than its normally expected resolution time [4] and an acute warning function of physiologic nociception is not present [5]. A pain is usually considered chronic if it lasts, or is present, for more than 3 to 6 months [6]. It has been estimated that chronic pain is present in approximately 20% of people globally [7–10] and accounts for 15% to 20% of all visits to the doctor’s office [11, 12]. Persons with chronic pain generally suffer from multiple physical and also psychological consequences of pain [13, 14]. Chronic pain increases the healthcare costs and cause losses in working hours due to absence from workplace [15, 16].

Negative experiences related to chronic pain can be considered the main life events, particularly among unemployed, but among employed too, because pain affects their working capacity [17]. Other emotional problems also occur, such as the feeling of futility and loss of control in everyday life or on working place [17]. People feel like victims, which is associated with the inability to accept its own situation regarding the pain [17]. Disorders of musculoskeletal system are the most common causes of chronic pain, leading to physical damage, limitations of working capacity, and unemployment in the long term [18]. According to the ICD 11, chronic musculoskeletal pain is defined as “persistent or recurrent pain that arises as part of a disease process directly affecting bone(s), joint(s), muscle(s), or related soft tissue(s)” [19]. For example, musculoskeletal disorders account for 53% of absences from working place in Norway and are the main reason for disability pensions [20]. Musculoskeletal pain thereby poses a huge economic burden [21].

It is known that the population of elderly people is rapidly growing worldwide [22]. Increasing age is often associated with complex health problems and is frequently combined with long-lasting musculoskeletal pain. The prevalence rate in communities of elderly people is even 60% [23, 24]. There is no significant difference in the prevalence of musculoskeletal pain between rural and urban populations. Social isolation is deemed to be more expressed in rural areas, which negatively impacts the perception of pain [25].

As reported by some authors, persons with chronic musculoskeletal pain are not satisfied with the effects of clinical treatment [26]. They believe that the health system, particularly doctors, does not take them seriously [26]. Doctors also believe that they are not able to do a lot for persons with chronic musculoskeletal pain and that the medical treatments have a little or no effect [26]. This is one of the main reasons why is self-care usually the first choice for the treatment of early disease symptoms [27]. “Self-care may be defined as the care taken by individuals towards their own health and well being, In practice it includes the actions people take to stay fit and maintain good physical and mental health; meet social and psychological needs; prevent illness or accidents; avoid unnecessary risks; care and self-medicate for minor ailments and long-term conditions; and maintain health and well being after an acute illness or discharge from hospital“[28].

The self-care implies the use of one or more medications not prescribed by a doctor, and procedures not supervised by the health service. It includes the use of herbal or pharmacological products, medications previously prescribed for similar cases, some medications from home pharmacy, or too short use of prescribed treatment [29]. It can also be defined as the use of medications as well as modern and traditional methods without consultations with the doctor (or other health care provider), or with no prescription and treatment supervision [30]. Considering various definitions and terms that describe the care about one’s own health, such as self-management, self-medication and self-treatment, the term self-care seems to be the most appropriate for this report since it covers more aspects and more different methods of care about patient’s own health investigated in this study.

Musculoskeletal pain is the most frequent reason for the use of complementary and alternative medicine (CAM) [31]. According to the investigation of Breivik H et al. [7] the most common self-care methods are the massage, physical therapy and acupuncture. In that study 38% of subjects reported that those methods were highly or very useful.

Asking for assistance of relatives as a strategy to cope with a pain can be useful, and stressful at the same time, because people who are in pain hardly accept the change of their role and asking for assistance [17]. The family is often a source of information and the transmission of traditional treatment methods and personal experiences from one generation to another [32]. Role of women in the family is the most important in this sense [33].

Self-care is related to increasing age [34, 35], which increases the risk of adverse events in self-care. The level of knowledge regarding methods and duration of treatment, and possible interaction of self-care with the use of already prescribed medications or the other form of treatment for another indication is lower in elderly population [36]. Younger generations, on the contrary, have better access to relevant information about self-care methods [34, 35].

Self-care of pain may have positive impact to pain alleviation and general disability, as well as to increase of psychological well-being such as lower level of stress, depression and anxiety [37].

Self-care studies are frequently carried out by the use of qualitative methods [38]. The aim of this study was to investigate experiences, needs and attitudes of patients regarding the self-care of chronic musculoskeletal pain, as a part of comprehensive care of chronic pain, ways to make decisions about the self-care, and expectations about the outcomes. We aimed to get an insight in the group of chronic pain patients that received the highest level of medical treatment of their chronic pain. At the same time, data regarding health care providers’ attitudes about patient’s self-care of musculoskeletal pain and its possible usefulness in relations to conventional medicine were collected.

In tis report, the first part of a larger mixed methods study aimed to test factors that are possibly associated with the good outcome of chronic pain treatment is presented. This part of the study explored the patient’s activation and his role in improvement of his condition.

## Methods

A qualitative, phenomenological approach was used, which allows understanding of certain phenomenon from the aspect of those who experienced it. It includes investigator’s intuition and open-minded acceptance of all adapted experiences and standpoints.

### Procedure

A series of a total of 35 semi-structured interviews, among them 15 with patients and 20 with health care providers, was carried out. Each subject was assigned an alias, to maintain the anonymity. By a method of audio-taped interview patients were asked about their understanding of the self-care phenomenon, their attitudes about self-care, and needs fulfilled in the self-care of chronic musculoskeletal pain (Table 1). The same method was used to ask health care providers about their attitudes regarding the patient’s self-care of musculoskeletal pain (Table 2). Interviews were carried out until saturation was attained. The saturation was defined and attained when answers in the last interviews were repeated and no new codes appeared in the analytical process [39]. All of interviews were carried out at the Outpatient Clinic for Pain Management, Sisters of Charity Clinical Hospital Centre Zagreb, Croatia, during the scheduled follow up or treatment visit. Conversations were transcribed verbatim and processed by a thematic analysis.

**Table 1:** List of questions for the semi-structured interview with patients

Study questions	Asked questions
Self-care attitude	What do you thing about self-care and what is your attitude about pain self-care?
Self-care decision	In what cases you decide to practice pain self-care?
	Please describe what influenced your decision to practice pain self-care
	• regarding the length of the pain
	• regarding the site and intensity of your pain
Self-care experiences	What do you expect from self-care?
	How satisfied are you with the outcome/result of self-care?
Self-care methods	Please describe your experiences with different methods of pain self-care?
Self-care information source	In what way and from whom you got the information about pain self-care?
Family tradition in self-care	Please describe the impact of family tradition to your decision to practice pain self-care.

**Table 2:** List of questions for the semi-structured interview with health care providers

Study questions	Asked questions
Self-care attitude	What do you think about patient’s self-care of pain and what is your attitude about this?
Self-care decision	In your opinion and experience, why patients decide to practice pain self-care?
Relation of self-care to clinical benefits in pain management	In what way is pain self-care related to successful outcome of medical management?

### Analysis

The thematic analysis was done according to Braun and Clarke [40] settings because it allows identification and analysis of topics within collected data and consequently the precise answer to study questions asked [40]. The aim of this qualitative analysis was to come to general conclusions or starting hypotheses for a quantitative study.

Following identification of key topics in line with the study questions and marking quotations containing terms relevant for the selected topic, the data were reduced based on the agreement between two independent investigators. Selected quotations were coded by an open codes which revealed characteristics derived from the text by an inductive method. Eventually a code list was formed. Based on code lists, related topics were grouped into categories/themes. Coding as a process developed relationships between main categories in line with the study questions and aim of the study.

In data interpretation the context of individual topics and codes, evident from the original text was respected. During the coding process, data from new interviews were continuously compared with data already analyzed. For the understanding and interpretation of phenomena studied, basic (raw) data were used eventually. Since the answers were coded by two independent investigators, the level of agreement for codes of each respective topic was determined by calculation of the agreement coefficient (Cohen’s kappa coefficient).

### Participants

Two subject groups were formed. Purposeful sampling method of participants of both groups was chosen according to the preselected criteria determined by the research question [41]. The participants were sought in the population of patients who received the highest level of medical pain management in the Outpatient Clinic for Pain Management so it was homogeneous in this characteristic, but we looked for the variety in patients’ age, gender, education and location of living (rural/urban) (Table 3). We included the patients with experience of chronic pain of different duration, different intensity and location to capture a wide range of perspectives on self-care and gain a greater insight into phenomena of self- care in this population from all aspects. A group of patients comprised 15 subjects, (until saturation) who were visiting the pain clinic. Before the start of interview patients were asked whether they practice self-care. If the answer was yes, the interview proceeded. Therefore the inclusion criteria were patients aged > 18 who practice self-care, any level of education and any place of residence, any type of chronic musculoskeletal pain and any duration of pain (> 3 month) (Table 3) while the exclusion criteria were age < 18 years, non-musculoskeletal pain and acute pain.

**Table 3:** Demographic characteristics of patient group

Characteristic		*n* = 15
Age		47.13 years (29–72)
Gender	M	5
	F	10
Employment status	Employed	13
	Retired	2
Educational level	Secondary school	10
	University or college degree	3
	Master’s or Ph.D. degree	2
Place of residence	Country	1
	Inhabited place with 5.000–50.000 inhabitants	3
	Town with > 50.000 inhabitants	11

The other group of 20 subjects (until saturation) comprised health care providers, experts for pain management or those experienced in management of patients in pain on primary care level. The subjects from health care provider group were nurses and doctors who differ in their age, gender, level of education, workplace, and professional experience (Table 4). Inclusion criteria for the health care provider sample were: specialist in anesthesiology, specialist in family medicine, nurse anesthetist, anesthesia technician, family medicine nurse, with variation in age, gender, level of education, workplace, and professional experience (Table 4). Although all health care workers see patients in pain, we limited our sample to the health care providers at two level of care that are most frequently involved in care of patients with chronic pain.

**Table 4:** Demographic characteristics of health care provider group

Characteristic		*n* = 20
Age		40,9 years (24–66)
Gender	M	5
	F	15
Profession	Specialist in anesthesiology	5
	Family doctor	1
	Resident in anesthesiology	2
	Nurse/technician at the anesthesiology department	11
	Nurse/technician in family medicine	1
Professional experience	Years of service	
	< 5	3
	5–9	4
	10–20	5
	> 20	8

Inclusion criteria were selected based on the aim of the study (Tables 1 and 2) and available literature data.

## Results

In total, 35 subjects entered the study: 15 patients from the Outpatient Clinic for Pain Management and 20 health care providers from the Department of Anesthesiology, Department of Surgery, and from general practice. Demographic characteristics of patient group are described in Table 3 and health care provider group in Table 4. The average age in patient group was 47 years. Most of them were employed, with prevailing secondary school education and the place of residence in a large urban area. The subjects from health care provider group were mostly nurses and technicians from the Department of Anesthesiology, Department of Surgery, Outpatient Clinic for Pain Management, and from general practice, as well as doctors – specialists or residents in anesthesiology. Their average length of service was 20 years.

From interviews, topics for each study groups were selected, representing their experiences in pain self-care.

### Duration and site of chronic musculoskeletal pain

Mean duration of the pain was 10.26 years (0.4–30.0 yrs). The shortest period of pain was 4 months and the longest one was 30 years.

The most common site of pain was lumbosacral region, followed by the cervical part of spine, pelvis and legs. An average intensity of pain evaluated by the pain assessment scale (0 – no pain, 10 – the worst possible pain) was 8 (6–10).

### Methods of self-care

The most common methods of pain self-care were the use of herbal products (various sorts of tea), and local administration of creams, gels and compresses. In addition, patients reported the use of acupuncture, yoga, meditation, exercise and walking. Patients often combine different methods of pain self-care.

### Themes, derived from the analysis

Three themes were recognized from analyzed interviews in each study group. Data were collected from the patient group revealed the following themes: positive aspects of self-care, need for self-care, and social aspects of self-care. Levels of agreement between two independent investigators for each theme were as follows:

1. Positive aspects of self-care, κ = 0.89
2. Need for pain self-care, κ = 0.71
3. Social aspects of self-care, κ = 0.90

Three themes recognized on the basis of data from the health care provider group were: positive aspects of self-care, need for self-care, and risks of self-care.

Levels of agreement for each topic were as follows:

1. Positive aspects of self-care, κ = 0.87
2. Need for pain self-care, κ = 1.0
3. Risks of self-care, κ = 0.69

After the first round of topic reading and recognizing, the topics were interpreted in the next round (Tables 5 and 6).

**Table 5:** Interpretation of patient topics

Positive aspects of self-care	Activation of patients (something that the patient can do for himself)
	Individuality of self-care:
	*-desire to control the pain*
	-healthy natural methods
	-integrated actions
	-psychological effect
	Easier to tolerate the pain
Need for pain self-care	Something should be done if the doctor is not visited
	Awkward and long-lasting pain
	Pain alleviating
	Improving functionality and quality of life
	Better effect of alternative and medicine combination
	Too much medication from conventional medicine
	Conventional methods exhausted
	Disappointment with conventional medicine
Social aspects of self-care	Information about self-care
	Tradition
	Experiences from others, sharing of experience
	Support from the environment

**Table 6:** Interpretation of health care provider topics

Positive aspects of self-care	Activation of the patient:
	*- Awareness*
	*- Care for her−/himself*
	*- Empowered patient*
	Psychological effect
	Lower burden for health services
	Lower consumption of analgesics
Need for self-care	The first form of treatment
	Avoiding of doctors (self-care preferred)
	Unavailability of doctors
	In mild conditions
	In acute conditions
Risks of self-care	Adverse effects
	Short-lived effect
	Mistakes in self-care
	Late diagnosis of pain causes
	Masking of symptoms
	Exaggeration

### Positive aspects of self-care

Results of this study reveal that the self-care is associated with patient’s activation where she/he does not simply wait for professional assistance but do something for himself, wishing to take the control over his/her pain. The self-care allows an individual treatment of pain because patients believe they know the best their bodies and their pain and are able on the best possible way assess what procedures are most appropriate for them. By self-care patients avoid a routine therapy which is the same for all of them. Patients tend to use healthy and natural treatment methods which are gentle and less aggressive than clinical ones, thus at the same time reducing the need for use of analgesics. The psychological effect of self-care is also emphasized. A power of belief that something is helpful may result in pain relief in the patient (at least in the short-term).

*„Why not? I am always open to all options. That what one believe, will surely help her/him”“, (P-13) (P – patient).*

*„Because I am somehow poisoned by all of those treatments, I avoid, really avoid… because I am full of chemistry, really.* “*(P-9).*

*„...I think that a human, a person can at most help itself...“(P-2).*

The health care providers agree with patients that self-care has a positive psychological effect and contributes to better psychic condition. Such a patient is easier to treat clinically. They also see higher activation in better informed patients. Unlike patients, health care providers think that self-care also reduce the burden of health services, while patients do not recognize or mention that.

*...those people put themselves a goal to take control over their pain as soon as possible, that they do not want to take a sick-leave, they wish to be vital, to have a quality of life, and in this respect the self-care makes sense.” (HP-17) (HP – health care provider).*

*“I support this, anyone knows its limits and needs the best.* “*(ZR-19).*

*„The self-care of pain has its place in the pain management and can be practiced until visit to doctor’s office, physicians are less burdened, and anything that is harmless can be tried.” (HP-1).*

### Need for self-care

The patients consider self-care essential in cases when something has to be done, for example, when the pain is intense and long-lasting but they do not visit the doctor, or when methods of clinical medicine are exhausted and they are disappointed with the treatment results. On the other hand, self-care can be an adjunct to clinical treatment (which is insufficient). While clinical medicine look for causes of pain, the patient try to help itself thereby improving its functionality and quality of life. The health care providers think that self-care is the first step in the pain management, particularly in some milder and acute conditions. They also think that patients prefer self-care to avoid the doctor, but there are also cases where the doctor is unavailable, or patients have to wait a long time for the examination or treatment.

*„Any self-care is welcome as long as it acts and I really think that all of us should take this way first, and then possibly, if this fails, go further or combine various treatment methods.* “*(P-14).*

*„When I simply exhausted all of options with my doctor, when even doctors did not know how to help me, then I had to go myself. (P-12).*

*„Well, because traditional, conventional medicine did not help me.” (P-2).*

*„…because to reach the therapy is very difficult. I think, you should wait for the physiatrist minimally for two months and then another two months for the therapy.* “*(P-15).*

*„It is good to start with some self-care prior to visit any institution.* “*(HP-13).*

### Social aspects of self-care

Patients talk to the family, friends and colleagues about their pain, thereby share their opinions and experiences. In addition to the Internet and various popular journals, those options are important source of information about methods of self-care. Family tradition plays a great role in transmission of positive experiences about the use of certain self-care methods from one generation to another.

*„All of this was through my family, meaning my parents, my mother. Well yes, I was told by my mom, she was told by my grandma, I think, or maybe my aunt.” (P-13);*

*„...Any available information was transmitted to me, and I transmit it further to my children.* “*(P-8).*

*„Well, through my friend.” (P-11).*

*„In the company where I am currently employed, a lot of people are dedicated to alternative treatment methods and they are really open to this so that experiences are frequently shared.* “*(P-14);*

*„Well, I do not know, most frequently, I am pretty often on the Internet and in this way I receive the alternative, really good tips are available, advices are healthy, all is natural..* “*(P-9); „Through the Internet, through various media.* “*(P-11); „…Then I read in the newspaper…* “*(P-14).*

### Risks of self-care

While patients do not see anything disputable in self-care processes, health care providers still emphasize that self-care may have adverse effects. Aberrances may occur in self-care which may result in symptom masking of certain serious conditions and in the late diagnosis of pain causes. Uninformed patients may exaggerate in the use of various self-care methods, thereby jeopardize their own health.

*„Masking of symptoms by certain medications, such as analgesics, may potentially be dangerous for the patient.” (HP-6).*

*“I think no, because they may come too late, when the pain is too heavy and then it is more difficult to treat it.* “(HP-11).

*„Patients persuade themselves that they get better with those various products and medications they use, thereby extending the period of treatment.“(ZR-10).*

## Discussion

The results or this study indicate that patients see themselves as individuals different from the others and therefore they expect individual treatment procedure. The same finding was reported by other authors too, because patients see the realization of their individuality and freedom of choice right in the self-care [42]. The holistic view on the health, ability to take the control over its own health, and the preference of natural treatment methods, as well as the concern about potential side effects of pharmaceutical products, are predictors of increased use of self-care [43–45]. The self-care is also encouraged by other factors, such as long waiting time for the examination and therapy. These findings are supported by the results of other studies. Patients are dissatisfied because they wait long for health services, are far from their physicians, and sometimes working hours of doctor’s office do not suit them [46, 47]. Reasons for self-care lie also in patient’s distrust of physicians, while, on the other hand, patients sometimes do not want to bother physicians, particularly if the cause of pain is already known [48]. The self-care is also a method of choice in cases where conventional medicine failed, or its methods have already been exhausted. The results of this study are in line with other reported ones. For example, Siapush et al. [49] demonstrated that dissatisfaction with the conventional medicine was caused by two factors. One factor was dissatisfaction with the outcome of medical treatment and the other one was dissatisfaction with the physician or another responsible health care provider.

While the attitude of subjects from a patient group to self-care was mostly positive, subjects from a health care provider group assumed that self-care can only be an adjunct to the treatment of pain. This finding is supported by other authors, such as Correa-Velez et al. [50] who emphasized that other methods were only an adjunct to conventional treatment methods, and were not contrary to them. Although self-care with medications is a practice in many developing countries, particularly by an urban and educated population [51], health care providers in our study assumed that self-care should be confined to alternative methods. Albeit the patient’s awareness of its need to make an effort for itself seems to be plausible, they warned to risks of self-care and its potential impact to diagnosis delay. They thought that patients undoubtedly should be well informed about methods used in the self-care. Non-awareness and use of various products in combination with prescribed medications may lead to interactions and adverse effects of self-care, such as intoxication or treatment failure [52–56].

Therefore health care providers in our study emphasized that self-care may carry potential risk for patient’s health and may mask symptoms of more serious diseases, with consequent prolongation of diagnosis. To take the responsibility for care of its health and to participate actively in the treatment process, patients still should possess certain proficiency and skills [57].

The family was in our study an important source of information about self-care of musculoskeletal pain, mostly when considering chronic musculoskeletal pain self-care that has been practiced in the family and transmitted from one generation to another. One of our results that should also be emphasized is a social aspect of self-care because people who suffer from similar difficulties talk to each other and share their experiences. Friends and colleagues from the workplace are therefore a common source of information about alternative self-care methods. However, when use of medications was considered, most of patients reported exclusively healthcare providers as sources of information, namely family doctors or anesthesiologist from the outpatient clinic for pain management. Similar conclusions were also drawn by other authors. According to results of Nagarajaiah et al. [36], important sources of information about self-care with medications are family members and relatives, followed by healthcare providers and pharmacists. A study carried out in Slovenia demonstrated that relatives and pharmacists were the most common sources of information and tips about self-care, and that self-care was more frequent among younger subjects and those with higher educational level [48, 58].

Our study demonstrated that the patients started with self-care by taking various sorts of tea and by a local administration of ointments, gels and compresses. Self-care methods using the tea, herbs and herbal products are well known [59]. The next methods of choice were acupuncture, chiropractic, yoga, meditation, exercise and walking. Other studies reported the acupuncture, chiropractic, message and yoga as most frequently used methods [37]. The psychological effect and power of belief that self-care will help have also their place in positive aspects of self-care, as confirmed by the similar studies [60]. The main results of our study emphasized the positive aspect of self-care, which was manifested in empowerment of the patient itself, where the self-care was understood as an individually adjusted procedure. Health care providers supported the self-care because it boosted the patient’s care about itself, but assumed that those procedures can be only an adjunct to the clinical treatment.

### Advantages and limitations of the study

The attitudes and experiences of patients and health care providers of various profiles were studied at the same time.

### Limitation

The patient sample was purposeful and not representative so that results cannot be translated to the whole population. We also did not expand the profile of health workers that are included in the treatment of chronic musculoskeletal pain especially specialists of physical medicine and physiotherapists.

## Conclusions

Self-care of musculoskeletal chronic pain is understood as an activity of empowered patient and should be used by health care providers to further include patient in his treatment and motivate him for self-help within the safety of official medicine. Health care providers support self-care of musculoskeletal pain as an adjunct to clinical treatment. They, however, assume that self-care acts as a placebo, with short-time effect to chronic musculoskeletal pain. Because of possible risk of self-care adverse effects (late diagnosis and true cause of pain establishment) patients should be well informed about the methods used. Patients understand self-care of musculoskeletal pain as an individually adjusted procedure and believe in its effectiveness. They lack individualized care from conventional medicine. Family tradition methods are commonly used in the self-care.

## Abbreviations

CAM: complementary and alternative medicine
HP: health care provider
P: patient
